# Use of dietary and performance-enhancing supplements among male fitness center members in Riyadh: A cross-sectional study

**DOI:** 10.1371/journal.pone.0199289

**Published:** 2018-06-21

**Authors:** Yazed AlRuthia, Bander Balkhi, Marwan Alrasheed, Ahmed Altuwaijri, Mohammad Alarifi, Huda Alzahrani, Wael Mansy

**Affiliations:** 1 Department of Clinical Pharmacy, College of Pharmacy, King Saud University, Riyadh, Saudi Arabia; 2 Pharmacoeconomics Research Unit, College of Pharmacy, King Saud University, Riyadh, Saudi Arabia; 3 Department of Pharmacology and Toxicology, College of Pharmacy, King Saud University, Riyadh, Saudi Arabia; 4 Department of Pharmacy, King Khalid University Hospital, Riyadh, Saudi Arabia; Case Western Reserve University School of Medicine, UNITED STATES

## Abstract

This study aimed to explore the health beliefs and patterns of dietary supplement usage among fitness center members. This cross-sectional study was conducted in four large indoor fitness centers in Riyadh, Saudi Arabia. This study involved male fitness center members aged ≥18 years with no speech or hearing disabilities. In-person interviews were conducted with fitness center members who agreed to participate using a newly developed questionnaire. Information on participants’ sociodemographics (e.g., age and education), smoking status, health status, exercise frequency, average time spent exercising, different supplements used, used supplements sources, and health beliefs regarding dietary supplements were obtained. A total of 445 fitness center members agreed to participate, and 198 of them reported taking dietary supplements. Most participants were between the age of 18 and 25 years (66%), had a college degree (74%), non-smokers (77%), healthy (84%), and perform exercise at least thrice weekly (52%) for at least 1 hour (63%). The percentage of participants who had favorable health views on dietary supplements was significantly higher among the supplement users than among the non-users (*P*<0.0001). Proteins, multivitamins, amino acids, and omega 3 fatty acids were the most commonly reported supplements used. Almost 30% of the supplement users reported buying them overseas, 28% online, 25% from a pharmacy or supplement store, 19% from a medical clinic, and 17% from peddlers. Public health campaigns are needed to educate the public on the potential harmful effects of supplements if purchased from an unofficial seller or taken without seeking medical advice before using them.

## Introduction

The demand for dietary supplements in the United States (US) has increased over the past 2 decades as people are becoming more health-conscious [1, 2]. The same upward trend in dietary supplements consumption was also noticed in the Middle East, especially among athletes [3–6]. According to the US Dietary Supplement Health and Education Act of 1994, which has amended the US Federal Food, Drug, and Cosmetic Act of 1938, dietary supplement is defined as any “product that contains dietary ingredient with purported additional nutritional value (e.g., supplement) to an individual’s daily diet” [7]. Although dietary supplements include products with proven nutritional values, such as multivitamins and minerals, many medicinal products with unproven nutritional or medicinal values are classified as dietary supplements, such as herbs and sexual performance-enhancing supplements [8].Today, many students and athletes in Saudi Arabia and the Middle East who consume dietary supplements have positive views on supplements in terms of their efficacy in improving their health and performance as well as their safety [3, 6]. This may arise from the widely spread belief that minerals, vitamins, and even herbal products are natural and harmless, which might not be the case all the time [8]. For example, abrupt withdrawal of high-dose vitamin D supplements may lead to a deficiency in the bioactive form of vitamin D, resulting in osteomalacia and muscle weakness [9]. Moreover, use of iron supplements for some individuals with no iron-deficiency anemia and certain genetic predisposition may lead to hemochromatosis and organ toxicity [10]. Therefore, taking dietary supplements is not appropriate for all individuals and these supplements should not be provided indiscriminately.

Moreover, the manufacturers of these supplements in the Saudi Arabia as well as in the US are only required by the Saudi Food and Drug Authority (SFDA) and the US Food and Drug Administration (USFDA), respectively, to adhere to the good manufacturing practices for dietary supplements to ensure that their products are safe, clean, and wholesome [11,12]. However, they are not required to provide any documentations, such as data from randomized clinical trials that prove the efficacy of their products as they claim [11, 12].

Conversely, many dietary supplements have been recalled from the market for being contaminated with prescription drugs and/or microorganisms [8, 12– 14]. Although the SFDA requires the manufacturers of dietary supplements to provide evidence that proves the safety and purity of their preparations besides examining a sample of the new products in the SFDA laboratories, some products were found to be contaminated with microorganisms after being approved and marketed [15]. For example, some weight loss products were found to be contaminated with aliphatic amine stimulants, such as 1,3-dimethylamylamine, which has been implicated with several cases of cerebral hemorrhage and cardiac arrest [16–19]. Moreover, some dietary products with performance-enhancing claims have been recalled from the US market after being contaminated with amphetamine analogs or sexual performance-enhancing prescription drugs, such as sildenafil [20, 21]. What is more concerning is that many athletes and fitness center members who consume dietary supplements are not aware of such dangers, especially if they buy such products from unlicensed sellers [4, 6, 13].

This study aimed to explore the pattern of use of dietary and herbal supplements as well as compare the perceptions and beliefs between users and non-users of these supplements among fitness center members in Riyadh, Saudi Arabia. In addition, the study aimed to explore the sources from where fitness center members buy these supplements.

## Materials and methods

### Study design

This cross-sectional, descriptive study was conducted in four large fitness centers in the city of Riyadh, Saudi Arabia. Male fitness center members, those aged ≥18 years, and those without speech or hearing disabilities were included in the study. Women were excluded owing to the cultural and religious restrictions in Saudi Arabia.

### Ethical consideration

The study was approved by the ethics committee of the College of Pharmacy at King Saud University. The legal approval to collect the data from the fitness centers was sought and obtained from Leejam Sports Company, which is the owner of the four large fitness centers that were surveyed. The collected data were kept in a safe and secure place according to the principles expressed in the Declaration of Helsinki.

### Sample size estimation

The minimum sample size was calculated using the Fisher’s exact test for inequality between two proportions (users vs. non-users of dietary supplements), with regard to their positive or negative beliefs on dietary supplements, at an alpha of 0.05, beta of 0.2, and medium effect size of 0.2 (e.g., 20% difference in the proportion between the two groups) [4]. The GPower^®^ 3.1 software was used to perform the power analysis [22].

### Study questionnaire

To explore the pattern of use of and beliefs regarding dietary supplements among fitness center members, a new questionnaire was developed by 4 members of the research team, who had previous experience in questionnaire development and validation, on the basis of the covered items of previously used and published questionnaires [1, 4, 23, 24]. The questionnaire consisted of 14 questions on the participants’ sociodemographics (e.g., age and education); chronic health conditions; regular medical checkups; frequency of exercise; average time spent on exercise daily; used dietary or performance-enhancing supplements, if any; and beliefs regarding dietary supplements (e.g., side effects, effect on exercise and health). Answers to these questions were recorded as “yes,” “no,” and “do not know.” Once the questionnaire was developed, it was sent to 3 external reviewers. The reviewers were a physician (general practitioner) who usually sees athletes for a yearly checkup, a clinical pharmacist who is active in patient counseling and education, and a researcher in social and behavioral sciences. In order to check the face validity, the reviewers were asked to rate the clarity of the questionnaire’s wording in an electronic form that rates each item of the questionnaire in a 4-point Likert scale (1 = unclear, 2 = somewhat clear, 3 = clear, 4 = very clear). Further, to check the content validity the reviewers were asked to provide their comments and suggestions regarding the relevance of the questionnaire to the beliefs about dietary supplements and to the study aims in a separate sheet that included each item of the questionnaire and a blank space next to it. The questionnaire was then revised based on the reviewers’ comments, and was sent back to them for further review. The revised version of the questionnaire was approved by all reviewers.

### Data collection

The eligible participants were asked to sign a consent form to ensure that they understood the purpose of this research as well as to assure them of the confidentiality and anonymity of any personal information before being interviewed for 10–15 min. Two pharmacy interns were involved in the data collection. The data collection started in January 2015 and ended in August 2015.

### Statistical analysis

Descriptive statistics were conducted using the chi-square test and Fisher’s exact test as appropriate to compare the proportion of the participants with favorable and unfavorable beliefs regarding dietary supplements between the two groups (users vs. non-users of dietary supplements). The significance level was specified at α = 0.05. All statistical analyses were performed using SAS version 9.2 (SAS Institute, Inc., Cary, NC, USA).

## Results

Table 1 presents the sociodemographic, medical, and lifestyle characteristics of the 198 participants who reported taking dietary supplements and 247 participants who denied taking any dietary supplements. Most of the participants were aged between 18 and 40 years, with no chronic health conditions, and had a relatively high level of education (e.g., an associate degree or above). Further, more than 75% of the participants were non-smokers with comparable results between the two groups. Conversely, most of them reported not having an annual medical checkup; however, a higher percentage of supplement users reported having an annual medical checkup (31.8% vs. 24.7%), exercising at least three times per week (83.3% vs. 69.4%), and spending at least an hour on exercise per day (81.3% vs. 65.4%) compared to non-supplement users.

**Table 1:** Sociodemographic, medical, and lifestyle characteristics of the participants.

Characteristics	Supplement users N *=* 198 (%)	Non-supplement users N = 247 (%)	Total population N = 445 (%)
**Age (y)**			
18–25	62.1	70.4	66.7
26–40	30.8	23.9	27
41–60	6.6	5.3	5.9
> 60	1.1	0	0.5
**Education**			
Less than high school	0.5	0	0.5
High school	12.1	20.0	16.5
College degree (e.g., associate or baccalaureate)	79.8	71	74.9
Graduate degree (e.g., master or PhD)	7.6	8.9	8.1
**Smoking status**			
Smoker	24.2	21.7	22.7
Non-smoker	76.8	78.3	77.3
**Any chronic diseases(e.g., asthma and diabetes)**			
No	91.9	92.7	84.6
Diabetes	3.0	0.4	1.6
Asthma	2.0	2.4	2.3
Gastrointestinal (e.g., IBS, IBD, GERD, and ulcer)	1.5	3.2	2.5
Hypertension	1.5	0	0.7
Hyperlipidemia	0	0.4	0.2
Multiple sclerosis	0	0.4	0.2
Epilepsy	0	0.4	0.2
**Regular medical checkup(e.g., every year)**			
No	68.2	75.3	72.2
Yes	31.8	24.7	27.8
**Frequency of exercise**			
1–2 days per week	16.7	30.5	24.5
3–5 days per week	58	47.6	52.2
6–7 days per week	25.3	21.9	23.4
**Average time spent on exercise daily**			
Less than 1 hour	18.7	34.6	27.5
1–2 hours	73.7	54.3	63
More than 2 hours	7.6	11.1	9.5

Note: Data are expressed as frequencies and percentages. IBS: Irritable Bowel Syndrome, IBD: Irritable Bowel Disease, GERD: Gastroesophageal Reflux Disease

The participants’ beliefs regarding dietary supplements are shown in Table 2. Significantly more non-users than supplement users believed that dietary supplements have side effects (52.2% vs. 43.4%; *P*<0.0001). By contrast, more supplement users than non-users believed that dietary supplements have positive effects on exercise performance (81.4% vs. 44.1%; *P*<0.0001), make them healthier (69.7% vs. 22.7%; *P*<0.0001), improve their stamina (64.7% vs. 31.2%; *P*<0.0001), boost their energy (71.2% vs. 48.9%; *P*<0.0001), help them cope with or overcome physical pain (42.9% vs. 20.6%; *P*<0.0001), and improve their ability to concentrate (42.4% vs. 21.5%; *P*<0.0001).

**Table 2:** Participants’ beliefs regarding dietary supplements.

Beliefs regarding dietary supplements	Supplement users N = 198 (%)	Non-supplement users N = 247 (%)	*P*-value
**Dietary supplements have side effects**			
Yes	43.4	52.2	<0.0001*
No	37.9	13.4	
Do not know	18.7	34.4	
**Dietary supplements have positive effects on exercise performance**			
Yes	81.8	44.1	<0.0001*
No	5.6	17.8	
Do not know	12.6	38.1	
**Dietary supplements make you healthier**			
Yes	69.7	22.7	<0.0001*
No	15.2	32.4	
Do not know	15.2	44.9	
**Dietary supplements improve your stamina**			
Yes	64.7	31.2	<0.0001*
No	17.7	18.6	
Do not know	17.7	50.2	
**Dietary supplements boost your energy**			
Yes	71.2	48.9	<0.0001*
No	17.7	12.9	
Do not know	11.1	38.1	
**Dietary supplements help you cope with or overcome physical pain**			
Yes	42.9	20.6	<0.0001*
No	32.8	26.7	
Do not know	24.2	52.6	
**Dietary supplements improve your ability to concentrate**			
Yes	42.4	21.5	<0.0001*
No	32.3	26.3	
Do not know	25.3	52.2	

*Significant difference (*P*<0.05)

**Fig 1:**
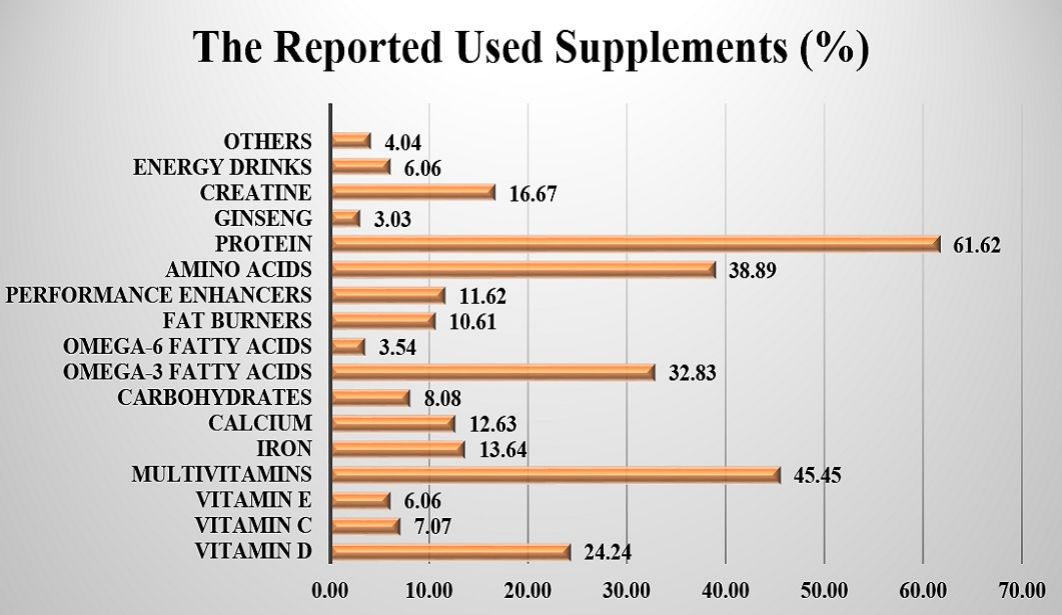
Dietary supplements used by the participants.

**Fig 2:**
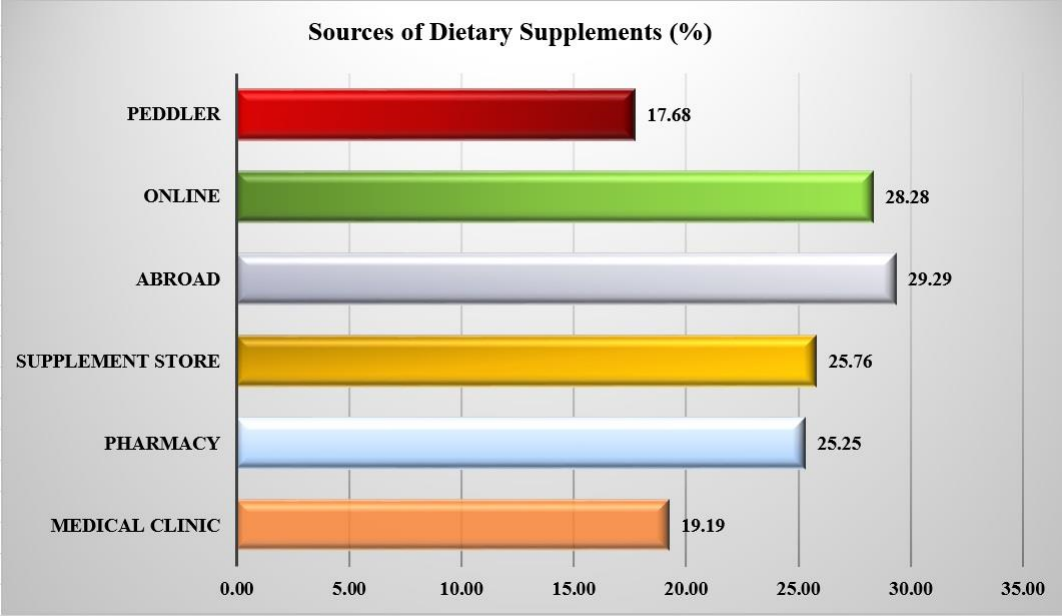
Sources of dietary supplements.

Fig 1 shows the different dietary supplements that were used by 44.5% of the participants. The five most commonly consumed supplements were protein (61.6%), multivitamins (45.5%), amino acids (38.9%), omega-3 fatty acids (32.8%), and vitamin D (24.2%). About 4.0% of the participants reported using dietary supplements, but did not recall their names; approximately 11.6% of them reported using dietary supplements with performance-enhancing claims, but did not mention their names. Out of the 198 dietary supplement users, 29.3% obtained the supplements from stores outside the country, 28.3% online, 25.8% from local dietary supplements stores, 25.3% from local pharmacies, 19.2% from local medical clinics, and 17.7% from unofficial sellers or peddlers (Fig 2).

## Discussion

The use of dietary supplements and performance enhancers is increasing among the Middle Eastern youth [1–4]. Almost half of the fitness center members who were included in this study reported using at least one dietary supplement. In addition to estimating the prevalence of the use of different dietary supplements among Saudi fitness center members, this study has compared positive and negative beliefs regarding dietary supplements between supplement users and non-users. Most of the participants who reported using dietary supplements had positive beliefs regarding dietary supplements. This is consistent with the findings of a previous study that explored Saudi athletes’ opinions on dietary supplements and the prevalence of their use among them [4]. Moreover, the proportion of participants with favorable views on dietary supplements was significantly higher among participants who reported using these supplements compared to non-supplement users. Furthermore, the proportion of participants who believed that dietary supplements have side effects was significantly lower among users of dietary supplements compared to non-supplement users. These comparisons were not reported or discussed among Saudi or Middle Eastern youth in previous studies.

Proteins, amino acids, multivitamins, vitamin D, and omega-3 fatty acids were the most commonly reported dietary supplements used among the participants, which is to a great extent similar to the findings of previously published studies [1–4]. Moreover, approximately 12% of the participants who reported using dietary supplements reported taking performance enhancers without providing any details of their names. These performance enhancers may contain illicit or prescription drugs, such as amphetamines, which can lead to serious consequences [18, 19]. Approximately 18% of the dietary supplement users reported buying their dietary supplements from unlicensed sellers or peddlers, which might be contaminated with microorganisms or contain unknown ingredients [7, 9, 10, 15]. Therefore, the SFDA and Ministry of Health should intensify their efforts to prevent the entry and sale of unlicensed dietary supplements.

### Limitations

Although this study was conducted in four fitness centers and was sufficiently powered, it was only conducted in one city. Therefore, the findings of this study cannot be applied to the general Saudi population. Further, recall bias is another issue, since some participants did not remember the name or the active ingredients of the dietary supplements they reported using on a regular basis. In addition, some important variables that might be associated with the use of dietary supplements among fitness center members, such as health literacy and income of the participants, were not collected.

## Conclusions

The results of this study should encourage public health researchers and health advocates to design and implement behavioral interventions through media campaigns and other means to educate the public on the benefits and risks of dietary supplements, especially of those used by athletes to enhance their performance. Those campaigns should also raise public’s awareness on the unexpected ingredients that have been found in dietary supplements purchased from unauthorized sellers. Further, the SFDA should work closely with the Ministry of Health to improve their oversight on the entry of unlicensed dietary supplements into the country and their inspection of pharmacies, medical clinics, and fitness centers that sell dietary supplements without having a license to sell such products.

## Supporting information

S1 De-identified Data (XLSX)

S1 Questionnaire Form (DOCX)
