# Supplementary material for: Use of dietary and performance-enhancing supplements among male fitness center members in Riyadh: A cross-sectional study
Source: PLoS One. 2018 Jun 21;13(6):e0199289. doi: 10.1371/journal.pone.0199289 (PMC6013215; doi:10.1371/journal.pone.0199289)
Supplement: S1 Questionnaire Form — (DOCX) [file pone.0199289.s002.docx]

The aim of this study is to explore the rate of food supplement utilization among fitness centers’ members as well as their beliefs about such supplements. The interview should not take more than 10-15 minutes from your time. .

1. **Age**

- 18-25
- 26-40
- 41-60
- >60

1. **Education:**
   - Less than high school.
   - High school.
   - College degree (e.g., associate or baccalaureate).
   - Graduate degree (e.g., master or PhD).
2. **Are you a smoker?**
   - Yes.
   - No.
3. **Do you suffer from any chronic diseases (e.g., asthma and, diabetes)?**
   - Yes.
   - No.
     1. **If Yes, Please mention them:** …………………………………………………………………………………………………………….
4. **Do you get regular medical checkups (e.g., every year)?**
   - Yes.
   - No.
5. **How often do you exercise?**
   - 1-2 days per week.
   - 3-5 days per week.
   - 6-7 days per week.
6. **How much time do you spend on average everyday exercising?**
   - Less than 1 hour.
   - 1-2 hours.
   - More than 2 hours.
7. **Do you believe that dietary supplements have side effects?**
   - Yes.
   - No.
   - I do not know.
8. **Do you believe that dietary supplements have positive effects on exercise performance?**
   - Yes.
   - No.
   - I do not know.
9. **Do you believe dietary supplements make you healthier?**
   - Yes.
   - No.
   - I do not know.
10. **Do you believe Dietary supplements improve your stamina?**
    - Yes.
    - No.
    - I do not know.
11. **Do you believe that dietary supplements boost your energy?**
    - Yes.
    - No.
    - I do not know.
12. **Do you believe that dietary supplements boost your energy?**
    - Yes.
    - No.
    - I do not know.
13. **Do you believe that dietary supplements improve your ability to concentrate?**
    - Yes.
    - No.
    - I do not know.

**If you are using any of the below supplements or performance enhancers please let us know:**

- Protein.
- Multivitamins.
- Amino acids.
- Omega-3 fatty acids.
- Omega-6 fatty acids.
- Vitamin D.
- Energy drinks.
- Creatine.
- Ginseng.
- Performance enhancers :…………………..
- Fat burners.
- Calcium.
- Iron.
- Carbohydrates.
- Vitamin E.
- Vitamin C.
- Others:……………….

**Where do you buy you supplements from?**

- Peddlers.
- Online.
- Abroad.
- Supplement stores.
- Pharmacy.
- Medical clinics.
